# AIDS-associated *Talaromyces marneffei* central nervous system infection in patients of southwestern China

**DOI:** 10.1186/s12981-020-00281-4

**Published:** 2020-05-26

**Authors:** Yu-Ye Li, Rong-Jing Dong, Samip Shrestha, Pratishtha Upadhyay, Hui-Qin Li, Yi-Qun Kuang, Xin-Ping Yang, Yun-Gui Zhang

**Affiliations:** 1 Department of Dermatology and Venereology, First Affiliated Hospital of Kunming Medical University, Kunming, 650032 China; 2 Yunnan Provincial Hospital of Infectious Disease/Yunnan AIDS Care Center (YNACC), Kunming, 650301 China; 3 Department of Medical Imaging, First Affiliated Hospital of Kunming Medical University, Kunming, 650032 China; 4 Institute of Infection and Immunology, Henan University & Center for Translational Medicine, Huaihe Clinical College, Huaihe Hospital of Henan University, Kaifeng, 475000 China

**Keywords:** AIDS, *T. marneffei*, Central nervous system infection, CSF, Antifungal therapy

## Abstract

**Background:**

The clinical and laboratory characteristics of AIDS-associated *Talaromyces marneffei* infection, a rare but a fatal mycosis disease of the central nervous system, remain unclear.

**Case presentation:**

Herein, we conducted a retrospective study of ten AIDS patients with cerebrospinal fluid culture-confirmed central nervous system infection caused by *Talaromyces marneffei*. All 10 patients were promptly treated with antifungal treatment for a prolonged duration and early antiviral therapy (ART). Among them, seven patients were farmers. Nine patients were discharged after full recovery, while one patient died during hospitalization, resulting in a mortality rate of 10%. All patients initially presented symptoms and signs of an increase in intracranial pressure, mainly manifesting as headache, dizziness, vomiting, fever, decreased muscle strength, diplopia or even altered consciousness with seizures in severe patients. Nine patients (90%) showed lateral ventricle dilatation or intracranial infectious lesions on brain CT. Cerebrospinal fluid findings included elevated intracranial pressure, increased leukocyte count, low glucose, low chloride and high cerebrospinal fluid protein. The median CD4^+^ T count of patients was 104 cells/μL (IQR, 36–224 cells/μL) at the onset of the disease. The CD4^+^ T cell counts of three patients who eventually died were significantly lower (W = 6.00, *p* = 0.020) than those of the patients who survived.

**Conclusions:**

The common clinical symptoms of *T. marneffei* central nervous system infection are associated with high intracranial pressure and intracranial infectious lesions. Earlier recognition and diagnosis and a prolonged course of amphotericin B treatment followed by itraconazole combined with early ART might reduce the mortality rate.

## Background

*Talaromyces marneffei* (*T. marneffei*, also known as *Penicillium marneffei*) is a regional opportunistic fungus that mainly causes life-threatening disseminated infection in acquired immunodeficiency syndrome (AIDS) that causes epidemics in southeast Asia [1] and south China [2, 3]. It is estimated that approximately 50,000 HIV-positive patients are infected with *T. marneffei* every year in high-risk areas [4], and the incidence is likely to increase every year [5]. The thermally dimorphic fungi mainly invade skin, lungs, liver, spleen, lymph nodes and the circulatory system. Infection involving the central nervous system (CNS) is extremely rare. Currently, only 22 cases of AIDS-associated *T. marneffei* CNS infection and two sporadic cases of non-AIDS patients with *T. marneffei* CNS infection have been reported worldwide [6–9]. These two non-AIDS patients suffered from *T. marneffei* CNS infections without any underlying immune deficiency diseases [7, 8]. Yet the mortality rate can be as high as 81% if diagnosis and treatment are delayed [9]. However, the clinical features of AIDS-associated CNS infection by *T. marneffei* are still unexplored. Here, we conducted a retrospective study on ten patients in southwestern China who were affected with AIDS-associated CNS infection by *T. marneffei* to characterize the clinical features and successful treatment of the disease.

## Case presentation

Ten patients were admitted to Yunnan Provincial Infectious Diseases Hospital from January 2009 to December 2015. *T. marneffei* fungi were isolated from CSF from all 10 AIDS patients. Sabouraud dextrose agar (SDA) was used for *T. marneffei* culture medium. We excluded other CNS infections (bacteria, viruses, and other fungi). These patients underwent smear examination, fungal culture and acid-fast bacillus culture, and no cryptococcus and tubercle bacillus were found in the blood and CSF. Serological experiments for syphilis and HSV-1/2 antibody in both blood and CSF were also negative. At the same time, we collected the data on clinical manifestations, imaging and laboratory examinations, therapeutic regimens and outcomes of all ten patients for analysis. This study was approved by the Ethical Committee of the First Affiliated Hospital of Kunming Medical University.

Categorical variables are presented as a percentage (%). The normal distribution data are expressed as the mean ± standard deviation ($\overline{\text{x}} \pm {\text{s}}$). Nonnormally distributed data are represented by median (interquartile range [IQR]). The CD4^+^ T cell counts of the deceased and surviving patients were nonnormally distributed, and the Mann–Whitney U test was used for comparison. Data analysis was performed using SPSS 22.0 software (SPSS Inc).

## Results

### Clinical features

The median age of the patients was 36.5 years (IQR [32.3–43.8]). Seventy percent of them were males. Out of ten patients, seven patients were farmers, two patients were unemployed, and one patient was a worker. Among them, eight patients acquired HIV by heterosexual transmission, one patient by homosexual transmission and one patient by intravenous transmission. The median duration of illness was 30 days (IQR [27.5–67.5]).

All ten patients had initial presenting symptoms of CNS infection, manifested as headache and dizziness in nine patients (90%), severe headache in five patients and dizziness in four patients. There were movement disorders of both lower extremities in six patients (60%); among them, three patients had lower limb dyskinesia, and three patients had bilateral lower limb fatigue. There were four patients (40%) with altered consciousness, two patients with drowsiness, one patient with unconsciousness and one patient with convulsions and coma. There were six patients with decreased muscle power of the lower limbs (60%), two patients with neck stiffness (20%) and projectile vomiting in three patients (30%). Unilateral hearing loss and unilateral ocular diplopia were present in one patient. High-grade continuous fever with temperature over 39 °C was present in five patients (50%). Skin lesions manifesting as a characteristic central necrotizing lesion combined with oral mucosal papules were present in two patients (20%). Six patients had been treated with ART before hospitalization (60%). Among them, five patients had been under treatment with ART for more than 2 years with regimen of AZT/3TC/NVP (EFV) or D4T/3TC/NVP and one patient had been under ART for 4 months with regimen of AZT/3TC/EFV (Table 1).

**Table 1:** Clinical features, treatments and outcomes of the 10 patients of AIDS-associated *T. marneffei* CNS infections

Patient	Gender	Age, (years)	Job	Duration of illness (days)	Test sample	Presenting symptoms	Presenting sign(s)	Initiation treatment and maintenance therapy	Hospitalization time	Initial ART time and regimen	Outcomes and followed up
1	Male	37	Farmer	30	CSF	Headache, nausea, vomiting, irritability, unconsciousness, fever	Neck stiffness	Amphotericin B for 38 days (0.5 mg/kg/day) followed by oral itraconazole (400 mg/day) for 10 weeks. Itraconazole (200 mg/day)	September 1, 2009	Mar 2007 AZT/3TC/NVP	Improved
2	Female	38	Farmer	60	CSF	Dizziness, eyes distention, limbs fatigue	–	Amphotericin B for 42 days (0.5 mg/kg/day) followed by oral itraconazole (400 mg/day) for 10 weeks. Itraconazole (200 mg/day)	January 20, 2010	Sep 2003 AZT/3TC/NVP	Improved
3	Male	36	Farmer	30	CSF and blood	Fever, right limb dyskinesia	A muscle power 4 out of 5 for right limbs, skin lesion and oral mucosal papules	Amphotericin B for 31 days (0.5 mg/kg/day) followed by oral itraconazole (400 mg/day) for 10 weeks. Itraconazole (200 mg/day)	May 31, 2100	Initial 28 days after hospitalization D4T/3TC/EFV	Improved
4	Male	47	Farmer	30	CSF	Headache, lower limb dyskinesia, hearing loss and diplopia	A muscle power 3 out of 5 for both lower limbs	Amphotericin B for 30 days (0.5 mg/kg/day) followed by oral itraconazole (400 mg/day) for 10 weeks. Itraconazole (200 mg/day)	April 16, 2013	Initial 29 days after hospitalization AZT/3TC/LPV/r	Improved
5	Male	46	Farmer	30	CSF	Dizziness, nausea, vomiting, drowsiness	A muscle power 3 out of 5 for right lower limb	Amphotericin B for 34 days (0.5 mg/kg/day) followed by oral itraconazole (400 mg/day) for 10 weeks. Itraconazole (200 mg/day)	January 7, 2015	Jul 2010 AZT/3TC/NVP	Improved
6	Male	30	Unemployment	60	CSF	Dizziness, lower extremity fatigue	A muscle power 4 out of 5 for right lower limb	Amphotericin B for 23 days (0.5 mg/kg/day) followed by oral itraconazole (400 mg/day) for 10 weeks. Itraconazole (200 mg/day)	January 23, 2015	Dec 2005 AZT/3TC/EFV	Improved
7	Female	34	Worker	90	CSF and urine	Fever, headache, Left upper limb dyskinesia	A muscle power 3 out of 5 for left upper limb	Amphotericin B for 40 days (0.5 mg/kg/day) followed by oral itraconazole (400 mg/day) for 10 weeks. Itraconazole (200 mg/day)	May 9, 2012	Initial 18 days after hospitalization AZT/3TC/EFV	Improved
8	Female	24	Farmer	20	CSF, blood and bone marrow	Fever, dizziness, weight loss, anorexia, convulsions and coma	Anemia, skin lesion, oral mucosal papules and enlargement of neck lymph node	Fluconazole for 6 days (400 mg/day)	March 7, 2013	–	Deterioration and dead
9	Male	33	Farmer	120	CSF	Fever, headache, drowsiness	–	Amphotericin B for 24 days (0.5 mg/kg/day) followed by oral itraconazole (400 mg/day) for 10 weeks	September 17, 2015	May 2015 AZT/3TC/EFV	Improved but died of unknown causes 1 year after discharge
10	Male	43	Unemployment	7	CSF	Limbs fatigue, headache, vomiting, urinary incontinence	Neck stiffness and a muscle power 1 out of 5 for both lower limbs	Fluconazole for 29 days (400 mg/day). Fluconazole (200 mg/day)	October 19, 2011	Oct 2007 D4T/3TC/NVP	Improved but died of unknown causes half a year after discharge

### CSF profiles and routine blood test

CSF examination revealed clear CSF in all patients and increased intracranial pressure (> 180 mmH_2_O) in eight patients (80%). The CSF leukocyte counts were increased (25 × 10^6^/L, IQR [13.8–113.5]). High protein concentrations were found in nine patients (123.7 mg/dL, IQR [66.1–234.5]), for which the normal concentration range was 15–45 mg/dL. The qualitative test of CSF protein was positive in nine patients (90%). The CSF glucose concentration was decreased (2.3 mmol/L, IQR [1.9–2.8]), for which the normal concentration range is 2.5–4.5 mmol/L. The median CSF glucose/serum glucose ratio in the CSF was 0.42 (IQR, 0.42–0.53). The CSF chloride was decreased (117.4 mmol/L, IQR [112.8–123.2]), for which the normal concentration range is 120–132 mmol/L. In addition to the positive CSF culture for *T. marneffei* in all patients, other body fluid sample cultures were also positive in three patients. Among them, one had positive results in both blood culture and bone marrow culture, one had a positive result in blood culture, and one patient had a positive result in urine culture. The median duration of CSF culture for *T. marneffei* was 11.5 days (IQR [6–14]) (Table 2).

**Table 2:** Laboratory findings of the 10 patients of AIDS-associated *T. marneffei* CNS infections

Peripheral blood cells analysis							CSF analysis					
Patient	CD4 (cells/μL)	CD8 (cells/μL)	WBC (×10^9^/L)	HB (g/L)	V-CSFP (mmH_2_O)	WBC (×10^6^/L)	NEU (%)	Lym (%)	CSF protein qualitative test	Sugar level (mmol/L) (normal, 2.5–4.5 mmol/L)	Protein level (mg/dL) (normal, 15–45 mg/dL)	Chloride level (mmol/L) (normal, 120–132 mmol/L)
1	220	725	5.23	119	290	104	28	72	2+	2	226	114
2	237	1051	3.1	129	180	7	–	–	1+	2.7	63.3	122.6
3	24	173	1.63	72	120	23	–	–	Negative	2.2	30.2	117.3
4	197	587	5	170	200	142	16	84	1+	2.3	128	125.1
5	80	169	11.1	134	225	16	–	–	2+	3	272	106.6
6	587	1123	5.49	169	> 330	27	–	–	1+	1.8	70	117.4
7	127	646	4.5	100	200	2	–	–	1+	2.7	67	130
8	10	96	1.61	76	150	21	–	–	1+	1.3	119.4	122
9	63	954	5.04	118	250	45	–	–	2+	2.1	260	114.1
10	40	132	4.32	166	320	170	27	73	3+	9.5	147.3	109.3

*CSF* cerebrospinal fluid, *WBC* white blood cell, *HB* hemoglobin, *Lym* lymphocyte, *NEU* neutrophil, *V-CSFP* ventricle-cerebrospinal fluid pressure

The CD8^+^ T cell counts were 616 cells/μL (IQR, 159–978 cells/μL), and the CD4^+^ T cell counts (104 cells/μL, IQR [36–224 cells/μL]) were decreased in all ten patients; the median CD4^+^/CD8^+^ ratio was 0.26 (IQR, 0.13–0.37). Among them, one patient died in the hospital, and two patients had improved clinically but died a year and half a year after discharge, respectively. The CD4^+^ T cell counts (40 cells/μL, IQR [10–63 cells/μL]) of the three patients who eventually deceased were significantly lower (W = 6.00, *p* = 0.020) than those of the patients who survived (209 cells/μL, IQR [115–325 cells/μL]). On blood tests, the median leukocyte count was 4.57 × 10^9^/L (IQR, 2.73–5.29 × 10^9^/L), of which three were leukopenic. The median hemoglobin count was 124 g/L (IQR, 94–166.75 g/L), and three patients were anemic (Table 2).

### Neuroimaging examinations

**Fig. 1:**
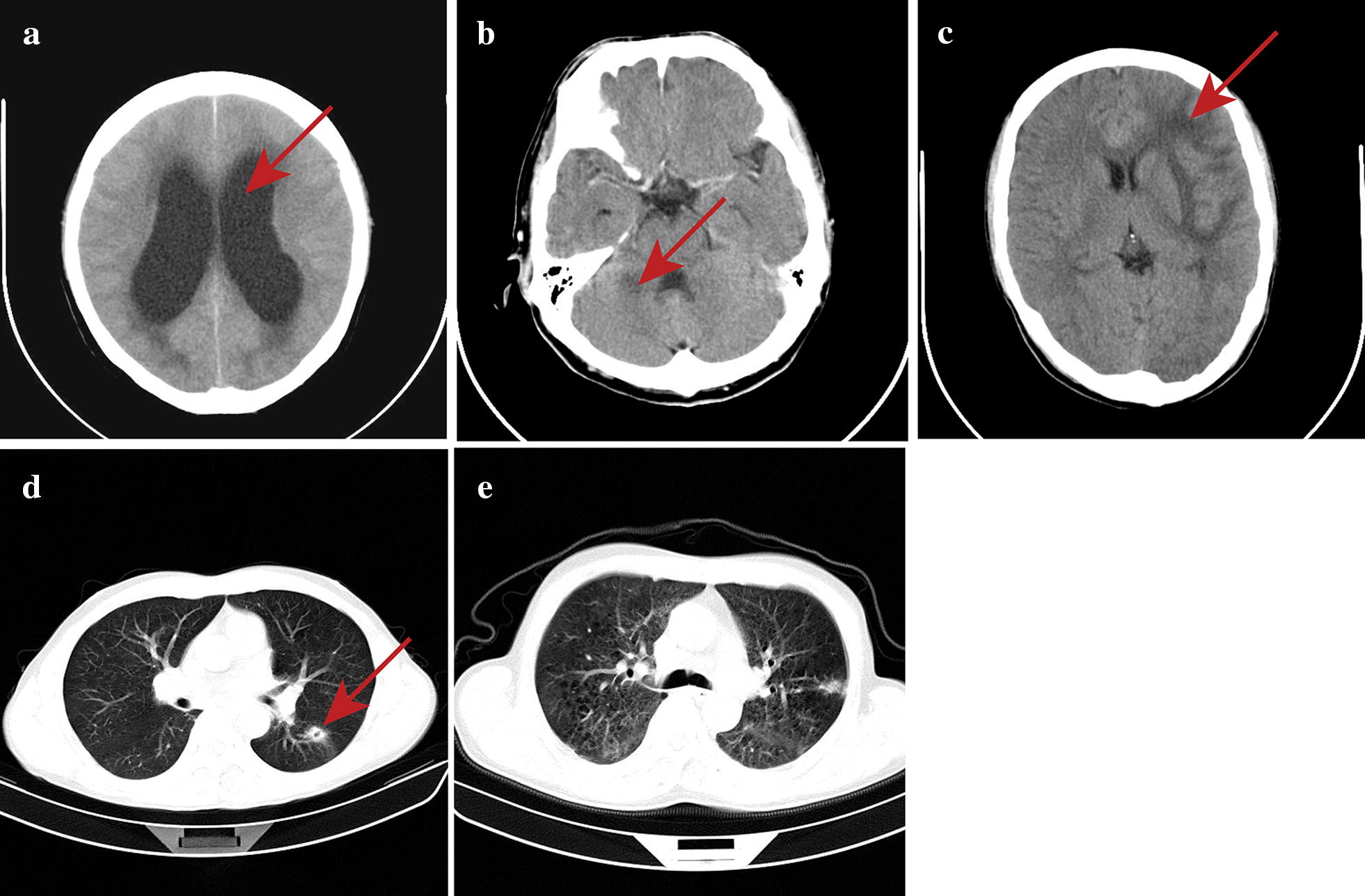
Brain and lung CT examination of the patients with AIDS-associated *T. marneffei* central nervous system infection. **a** Bilateral lateral ventriculomegaly: the bilateral lateral ventricles were dilated, and the brain parenchyma was slightly compressed bilaterally (arrow). **b** Patchy low-density shadow in the right cerebellar hemisphere in contrast enhanced CT: flaky low-density shadow on the right cerebellar hemisphere with unclear boundaries; the lesion was approximately 2 cm in size. There was no enhancement of the lesions after the use of contrast media (arrow). **c** Multiple intracranial infectious lesions with cerebral edema: parenchymal swelling in the left frontal lobe, insula and basal ganglia, cerebral sulcus thinning, extensive edema in the white matter parenchyma of the frontal lobe, showing low density (arrow). **d** Left pulmonary nodules with voids: high-density nodules in the dorsal segment of the left lower lobe, approximately 2 cm in diameter, with unclear boundaries and a cavity within the lesion (arrow). **e** Interstitial pneumonia: diffuse ground-glass opacity of bilateral lungs, patchy high-density shadow of the subpleural area in the posterior segment of apex of left lung, with thickening of the adjacent pleura

In addition to symptoms and signs of central nervous system infection and abnormal CSF and hematological profile, nine patients had abnormal brain CT scans (90%), four patients had lateral ventricle dilatation, four patients had intracranial infectious lesions and one patient had intracranial infectious lesion along with cerebral ventricular dilatation. CT abnormalities in the lungs were observed in five patients (50%), four patients had pulmonary nodules, and one patient had interstitial pneumonia (Fig. 1).

### Treatment and outcome

All ten patients were started with initial therapy, eight patients were treated with amphotericin-B (AmB) (0.5 mg/kg/day), and the mean duration of initial therapy by AmB was 32.33 ± 6.72 days, which was extended for more than 2 weeks until the CSF fungus culture was negative. The initial treatment was then followed by oral itraconazole (400 mg/day) for 10 weeks. One patient who had convulsions and coma before hospital admission died during the course of treatment with fluconazole (400 mg/day) empirical antifungal treatment on the 7th day in the hospital. One patient who was treated with fluconazole for initial and maintenance therapy died after half a year of discharge. The initial therapy was then followed by chronic maintenance therapy with oral itraconazole (200 mg/day), which was given until CD4 counts were more than 200 cells/μL. One patient died of unknown causes who had been treated initially with AmB for 24 days and oral itraconazole for 10 weeks but had not undergone maintenance treatment. Patients who had not received ART before the onset of disease were all started on ART within 1 month of hospital admission. The median duration of hospital stay was 40 days (IQR [25–54]). The remaining 7 patients followed up until April 2019 had no recurrence of the disease. With regular ART, the CD4^+^ T cell count gradually increased to 498 ± 216 cells/μL. The viral load in the remaining patients was below the detection limit.

## Discussion and conclusions

Disseminated *T. marneffei* infection mainly involves the circulatory, respiratory and digestive systems, resulting in common clinical symptoms, including fever, cough, abdominal pain, weight loss, anemia, and cutaneous lesions [10]. However, CNS involvement is rare. In this study, besides the common symptoms such as fever, anemia and skin lesions, all the patients predominantly showed initial presenting symptoms or manifestations of CNS infection, including headache, vomiting, dizziness and dyskinesia or fatigue. Severe patients presented with altered consciousness, such as drowsiness, loss of consciousness, convulsions or coma. Other signs the patients had included decreased lower limb muscle strength and neck rigidity, which were associated with intracranial hypertension. We reviewed previous sporadic cases, which also showed the typical symptoms of CNS *T. marneffei* infection among the patients with or without HIV infection [6–8]. Wu et al. [8] reported a very rare case of a non-AIDS patient with *T. marneffei* CNS infection who had hearing loss and diplopia, which may be due to yeast infiltration of the optic and acoustic nerves; they suggested that if patients have hearing loss, this disease also needs to be considered. However, Le et al. [9] reported that the symptoms among 21 patients under their study were mostly fever (90%), anorexia (57%), fatigue (52%), cough (33%), and diarrhea (29%) and that only 10 cases (47%) showed initial symptoms of acute CNS infection, while symptoms of increased intracranial pressure and meningismus were absent in the others, which was very different from our study.

The common clinical manifestations of CNS fungal infections vary from chronic meningitis or meningoencephalitis or abscesses to a fungal ventriculitis [11]. We found that the majority of our patients had abnormal neuroimaging findings, mainly manifested as ventricular dilatation and intracranial infectious lesions, and half of them had pulmonary lesions. Combined with other reports of bilateral lateral ventricular hydrocephalus and intracranial infection lesions [6–8], we are assured that the central nervous system lesions caused by *T. marneffei* are mainly meningitis. The existing study found that ventriculomegaly is a frequent finding in the neuroimages of patients with advanced HIV/AIDS disease due to HIV encephalopathy with cerebral atrophy [12]. However, the pathophysiology of *T. marneffei* invading the blood–brain barrier is still unclear. Additionally, we observed elevated CSF intracranial pressure, increased leukocyte count, low CSF glucose, low CSF chloride and high CSF protein levels in our study, which were consistent with previous case reports [6, 8, 9]. Low CSF glucose level may be related to glucose utilization by microorganisms, and the degree of hypoglycemia is directly correlated with the inflammatory process [13]. A previous study speculated that the low CSF glucose level can be used as a marker for the diagnosis of microbial meningitis [14]. Our results showed that the CSF profile of AIDS-associated *T. marneffei* CNS infection is extremely similar to those of other types of fungal meningitis, especially cryptococcal meningitis. This condition can be easily confused since cryptococcal meningitis is also a common opportunistic infection in HIV patients.

*Talaromyces marneffei* mainly infects immunocompromised individuals such as AIDS patients, particularly when individuals have CD4^+^ T cell counts < 50 cells/μL [15]. Studies have found that CD4^+^ T cells were usually below 50 cells/μL in HIV-associated cryptococcal meningitis and that some severe cases even had levels below 30 cells/μL [16, 17]. However, we observed that the median CD4^+^ T cell counts were 104 cells/μL, and three patients’ CD4^+^ T cells were above 200 cells/μL. Patients with relatively high levels of CD4^+^ T cells who also present with *T. marneffei* CNS infection may be due to subclinical infection of *T. marneffei* when patients’ CD4^+^ T cell counts was low, these patients develop unmasking immune reconstitution inflammatory syndrome (unmasking IRIS) after initial ART [18]. We assured that patients in epidemic areas have relatively higher CD4^+^ T cells, they should also be alerted to *T. marneffei* CNS infection. In addition, patients No. 8, 9, and 10, who eventually deceased, had very low CD4^+^ T cell counts (10, 63 and 40 cells/μL, respectively), especially patient No. 8, who died due to deterioration during hospitalization. Le et al. [9] also reported that the median CD4^+^ T cell counts of dead patients were 11 cells/μL in AIDS-associated *T. marneffei* CNS infection. We supposed that low CD4^+^ T cell counts may be associated with high mortality.

Currently, the recommended treatment for AIDS-associated *T. marneffei* non-CNS infection is intravenous AmB for 2 weeks followed by oral itraconazole (400 mg/day) for 10 weeks, then followed by oral itraconazole (200 mg/day) as maintenance therapy, with ART started as soon as possible [19, 20]. Considering the penetration of the blood–brain barrier, the concentration of the drug in the blood–brain and the long duration of illness, most patients initiated and prolonged anti-fungal therapy with AmB and itraconazole for maintenance therapy. However, patient No. 8, who died during hospitalization before confirmed *T. marneffei* infection, had received empirical antifungal fluconazole therapy. Patient No. 9 had improved clinically but died after a year of discharge, he only received initial treatment and lacked maintenance treatment. Patient No. 10, who had been treated with fluconazole for both initial and maintenance therapy, had improved clinically, but eventually died after half a year of discharge. Both patients were more likely to die of recurrence of *T. marneffei* infection. AmB has fungicidal activity and is superior to fluconazole, itraconazole, and voriconazole against *T. marneffei,* although they can treat HIV-associated fungus infection effectively [21–23]. Moreover, AmB or liposomal amphotericin B (LipAmB) are the most common and successful antifungal drugs for CNS fungal infections, although these drugs have generally low or unmeasurable levels in the CSF. A study recommended that an AmB/LipAmB + 5FC regimen should be used as a first choice to treat cryptococcal meningitis in patients [24]. In our study, we also suggest that AmB is preferred for initial therapy and extension of the initial course until cerebrospinal fluid fungus cultures become negative, which can be more than 2 weeks as recommended in cases of cryptococcal meningitis [19]. Then, maintenance treatment should be continued with itraconazole until CD4^+^ T levels increase to more than 200 cells/μL. Then, there arises a thought-provoking question about when to start ART. Delayed ART may affect fungal clearance and immune system recovery. Early initiation of ART within the first 2 weeks of antifungal therapy may paradoxically induce IRIS in cases of cryptococcal meningitis [25–27]. Thus, the initiation of ART should be based on fungal load, CD4^+^ T cell counts and the CSF fungal culture report after 2 weeks of therapy [28, 29]. Thus, a more rational management of HIV/AIDS coinfection with *T. marneffei* CNS infection needs to be further investigated.

Last, in our study, 7 out of 10 patients were farmers from different areas. The occupational relationships with the disease have not been reported by any other studies. The exact etiology of *T. marneffei* infection is not yet clear. Thus far, bamboo rats are believed to be the only reservoir of *T. marneffei.* Chariyalertsak et al. [30] identified agricultural exposure to soil during the rainy season as an important risk factor for *T. marneffei* infection. Therefore, farmers have high probability of contact exposure to the soil-exposed *T. marneffei* fungal spores and soil-burrowing bamboo rats.

To the best of our knowledge, we are the first to comprehensively report the clinical manifestations and laboratory characteristics of AIDS-associated *T. marneffei* CNS infection. In HIV/AIDS and *T. marneffei* epidemic areas, when patients exhibit symptoms and signs of acute nervous system infection or even show any neurological symptoms, they should be investigated for CSF fungal culture and given a brain imaging examination to make a comprehensive diagnosis. In the routine investigation of CSF, viral investigations, bacterial culture and fungus culture should be carried out under different temperature and time durations, as *T. marneffei* grows slowly in culture media. Notably, it is also necessary to rule out cryptococcal meningitis and other similar conditions since the symptoms of these diseases are similar but the treatment methods are different. Prolonged course of AmB treatment followed by itraconazole as recommended for *T. marneffei* non-CNS infection combined with early initial ART might reduce the mortality rate.

## Data Availability

Data sharing not applicable to this article as no datasets were generated or analysed during the current study.

## References

[1] Supparatpinyo K, Khamwan C, Baosoung V, Nelson KE, Sirisanthana T (1994). Disseminated *Penicillium marneffei* infection in southeast Asia. Lancet.

[2] Li X, Yang Y, Zhang X, Zhou X, Lu S, Ma L (2011). Isolation of *Penicillium marneffei* from soil and wild rodents in Guangdong, SE China. Mycopathologia.

[3] Dong RJ, Zhang YG, Zhu L, Liu HL, Liu J, Kuang YQ (2019). Innate immunity acts as the major regulator in *Talaromyces marneffei* coinfected aids patients: cytokine profile surveillance during initial 6-month antifungal therapy. Open Forum Infect Dis.

[4] Armstrong-James D, Meintjes G, Brown GD (2014). A neglected epidemic: fungal infections in HIV/AIDS. Trends Microbiol.

[5] Hu Y, Zhang J, Li X, Yang Y, Zhang Y, Ma J (2013). *Penicillium marneffei* infection: an emerging disease in mainland China. Mycopathologia.

[6] Lei Y, Qiu Y, Zhang JQ, Liu GN, Deng JM, He ZY (2015). A case report of *Penicillium marneffei* involving the central nervous system and literature review. Chin J Pract Intern Med.

[7] Liu GH, Gu YY, Jiang GH, Mo MC, Chen GQ (2012). Disseminated Marniffe Penicillium involving the central nervous system: a case report and literature review. J Clin Exp Pathol.

[8] Wu LL, Zhong JM, Mao DA (2014). A report case of hearing loss caused by *Penicillium marneffei* combined with central nervous system infection. Chin J Contemp Pediatr.

[9] Le T, Huu Chi N, Kim Cuc NT, Manh Sieu TP, Shikuma CM, Farrar J (2010). AIDS-associated *Penicillium marneffei* infection of the central nervous system. Clin Infect Dis.

[10] Kawila R, Chaiwarith R, Supparatpinyo K (2013). Clinical and laboratory characteristics of penicilliosis marneffei among patients with and without HIV infection in Northern Thailand: a retrospective study. BMC Infect Dis.

[11] Gavito-Higuera J, Mullins CB, Ramos-Duran L, Olivas Chacon CI, Hakim N, Palacios E (2016). Fungal infections of the central nervous system: a pictorial review. J Clin Imaging Sci.

[12] Udgirkar VS, Tullu MS, Bavdekar SB, Shaharao VB, Kamat JR, Hira PR (2003). Neurological manifestations of HIV infection. Indian Pediatr.

[13] Shrikanth V, Salazar L, Khoury N, Wootton S, Hasbun R (2015). Hypoglycorrhachia in adults with community-acquired meningitis: etiologies and prognostic significance. Int J Infect Dis.

[14] Baud MO, Vitt JR, Robbins NM, Wabl R, Wilson MR, Chow FC (2018). Pleocytosis is not fully responsible for low CSF glucose in meningitis. Neurol Neuroimmunol Neuroinflamm.

[15] Skoulidis F, Morgan MS, MacLeod KM (2004). *Penicillium marneffei*: a pathogen on our doorstep?. J R Soc Med.

[16] Nguyen MH, Husain S, Clancy CJ, Peacock JE, Hung CC, Kontoyiannis DP (2010). Outcomes of central nervous system cryptococcosis vary with host immune function: results from a multi-center, prospective study. J Infect.

[17] Bratton EW, El Husseini N, Chastain CA, Lee MS, Poole C, Sturmer T (2012). Comparison and temporal trends of three groups with cryptococcosis: HIV-infected, solid organ transplant, and HIV-negative/non-transplant. PLoS ONE.

[18] Chang CC, Sheikh V, Sereti I, French MA (2014). Immune reconstitution disorders in patients with HIV infection: from pathogenesis to prevention and treatment. Curr HIV/AIDS Rep.

[19] Kaplan JE, Benson C, Holmes KK, Brooks JT, Pau A, Masur H (2009). Guidelines for prevention and treatment of opportunistic infections in HIV-infected adults and adolescents: recommendations from CDC, the National Institutes of Health, and the HIV Medicine Association of the Infectious Diseases Society of America. MMWR Recomm Rep.

[20] Nor-Hayati S, Sahlawati M, Suresh-Kumar C, Lee KC (2012). A retrospective review on successful management of *Penicillium marneffei* infections in patients with advanced HIV in Hospital Sungai Buloh. Med J Malays.

[21] Lei HL, Li LH, Chen WS, Song WN, He Y, Hu FY (2018). Susceptibility profile of echinocandins, azoles and amphotericin B against yeast phase of *Talaromyces marneffei* isolated from HIV-infected patients in Guangdong, China. Eur J Clin Microbiol Infect Dis.

[22] Le T, Kinh NV, Cuc NTK, Tung NLN, Lam NT, Thuy PTT (2017). A trial of itraconazole or amphotericin B for HIV-associated talaromycosis. N Engl J Med.

[23] Rx Y, Wn S, Xm Q, Jz B (2007). Analysis of fungus infection and drug sensitivity in AIDS patients. Guangdong Med.

[24] Xu L, Zhang X, Guo Y, Tao R, Dai X, Yang Z (2019). Unique clinical features of cryptococcal meningitis among Chinese patients without predisposing diseases against patients with predisposing diseases. Med Mycol.

[25] Perfect JR, Dismukes WE, Dromer F, Goldman DL, Graybill JR, Hamill RJ (2010). Clinical practice guidelines for the management of cryptococcal disease: 2010 update by the infectious diseases society of america. Clin Infect Dis.

[26] Perfect JR, Bicanic T (2015). Cryptococcosis diagnosis and treatment: what do we know now. Fungal Genet Biol.

[27] Boulware DR, Meya DB, Muzoora C, Rolfes MA, Huppler Hullsiek K, Musubire A (2014). Timing of antiretroviral therapy after diagnosis of cryptococcal meningitis. N Engl J Med.

[28] Jarvis JN, Bicanic T, Loyse A, Namarika D, Jackson A, Nussbaum JC (2014). Determinants of mortality in a combined cohort of 501 patients with HIV-associated Cryptococcal meningitis: implications for improving outcomes. Clin Infect Dis.

[29] Franco-Paredes C, Chastain DB, Rodriguez-Morales AJ, Marcos LA (2017). Cryptococcal meningoencephalitis in HIV/AIDS: when to start antiretroviral therapy?. Ann Clin Microbiol Antimicrob.

[30] Chariyalertsak S, Sirisanthana T, Supparatpinyo K, Praparattanapan J, Nelson KE (1997). Case-control study of risk factors for *Penicillium marneffei* infection in human immunodeficiency virus-infected patients in northern Thailand. Clin Infect Dis.

